# Fluorescence control of chitin and chitosan fabricated *via* surface functionalization using direct oxidative polymerization†

**DOI:** 10.1039/c8ra00287h

**Published:** 2018-02-13

**Authors:** Thien An Phung Hai, Ryuichi Sugimoto

**Affiliations:** School of Environmental Science and Engineering, Kochi University of Technology Miyanokuchi, Tosayamada Kami Kochi 782-8502 Japan an.phthien@gmail.com sugimoto.ryuichi@kochi-tech.ac.jp

## Abstract

The copolymer of 3-hexylthiophene (3HT) and fluorene (F) was directly grafted onto chitin and chitosan using FeCl_3_ as an oxidant. The properties of the grafted chitin/chitosan were characterized by Fourier transform infrared (FT-IR) spectroscopy, UV-Vis spectroscopy, fluorescence spectroscopy, X-ray diffraction analysis, thermogravimetric analysis (TGA), transmission electron microscopy-energy dispersive X-ray spectroscopy, and quantum yield measurements. The UV-Vis absorption peaks of the chitin/chitosan grafted with 3-hexylthiophene and fluorene copolymer were increasingly blue-shifted upon increasing the fluorene content and the red-shifted emission of the grafted chitin/chitosan were controlled by varying the monomers feed of the 3HT/F units. The hypsochromic and bathochromic shifts of chitin/chitosan were ascribed to the (3HT/F) moieties grafted to their surface. The quantum yield of grafted chitin/chitosan increased upon increasing the fluorene content. The TGA and XRD analysis revealed that the thermal stability and crystallinity of chitin/chitosan decreased upon grafting the copolymer of fluorene and 3-hexylthiophene. This article represents a simple route towards the surface modification of chitin and chitosan using conducting copolymers, providing multicolor chitin and chitosan *via* a one-step reaction.

## Abstract 2

The copolymer of 3-hexylthiophene (3HT) and fluorene (F) was directly grafted onto chitin and chitosan using FeCl_3_ as an oxidant.

## Graphical abstract

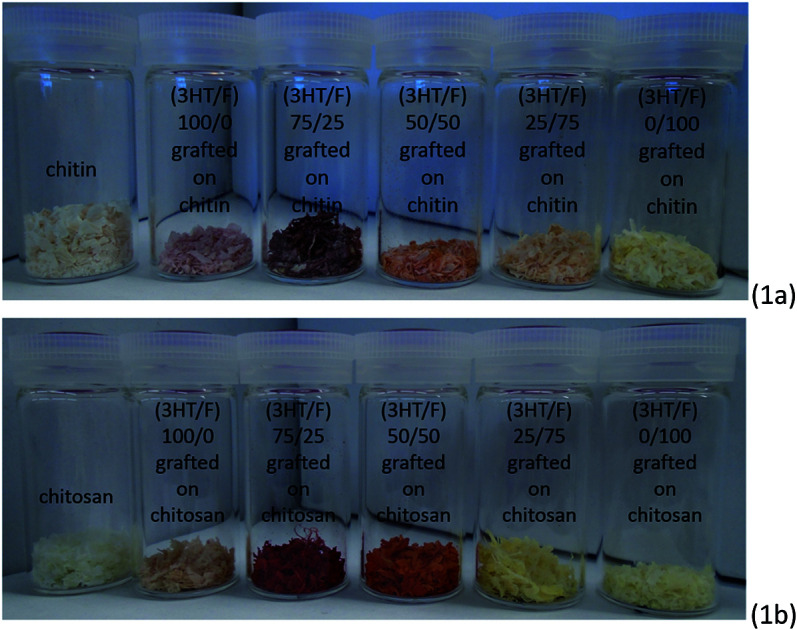

## 1. Introduction

Chitin and chitosan are crucial biopolymers with special chemical reactivity and physical properties. They are used in diverse biomedical applications including drug delivery systems, tissue engineering, wound dressings, antibacterials, cancer diagnosis and electrochemical sensor design.^1–7^ During the last two decades of research on novel functionalized and sustainable chitin/chitosan-based materials, various efforts have been carried out to enhance the physical and chemical properties of these polysaccharides using different modification techniques. Chitin and chitosan have also been grafted with polystyrene (PS) *via* a free radical mechanism using ammonium persulfate (APS).^8,9^ The surface modification of chitin nanocrystals with poly(3-hydroxybutyrate-*co*-3-hydroxyvalerate) (PHBV) *via* chloridization improved the lipophilicity of chitin nanocrystals.^10^ The high capacity absorbance of chitin fibers functionalized with a uranyl-selective amidoxime group used for the extraction of uranium from seawater has been reported.^11^ The hydrophobic of the modified chitin surface leads to the highly effective separation of oil from water.^12^ An amphiphilic chitosan surface has been achieved by grafting hydrophilic polyacrylamide and hydrophobic polystyrene to allow reversible switching in polar and non-polar media.^13^ An improvement in the hydrophilic properties of chitin nanofibers *via* graft polymerization with acrylic acid has also been accomplished.^14^

“Grafting through”,^15–17^ “grafting onto”^18–20^ and “grafting from”^21^ are three frequently used strategies for synthesizing graft polymers. Recently, advances in the cooperation between the conjugated polymers and biomaterials have led to new interest in engendering materials with novel functionalities, further opening the scope of applications to biosensors, bioelectronics, tissue engineering, and biofuel cells.^5,22–24^

The composites of chitin/chitosan and conjugated polymers prepared *via* a simple oxidative polymerization with ammonium persulfate have been applied as sensor materials and electrodes in polymeric batteries.^25–27^ Improving the optical properties of biomaterials such as cellulose and polysaccharide is now the focus of attention due to its potential applications. A chitin nanocrystal conjugated with both fluorescent dye and carbohydrate ligand has been developed for biorecognition applications.^28^ Self-assembly conjugated polymers encapsulated in chitosan-*graft*-oleic acid with visually noticeable fluorescence response from blue to red have been reported as an alternative method for the detection of aliphatic biogenic amines.^29^ A Diels–Alder cycloaddition and thiol-Michael “click” reaction has been developed to graft a fluorescein derivative and coumarin onto cellulose nanofibrils (CNF), yielding multicolor CNF.^30^

The oxidative polymerization of thiophene was discovered three decades ago and is now widely used due to its facile polymerization.^31–36^ In our previous reports, the surface modification of cellulose and polysaccharides with conjugated polymers has been successfully accomplished *via* oxidative polymerization using FeCl_3_.^37–39^ In this study, copolymers composed of two different conjugated polymers, including 3-hexylthiophene and fluorene were applied for the surface modification of chitin and chitosan. It is noticeable that multicolor materials can be manipulated by controlling the monomer composition of 3-hexylthiophene and fluorene.

## 2. Materials and experiments

### 2.1 Chemicals

Chitin and chitosan flakes were purchased from Tokyo Chemical Industry Ltd (TCI). Commercial chitosan from TCI had a degree of deacetylation (DD) of min. 80% and medium molecular weight with a viscosity of 0.5% chitosan in 0.5% acetic acid solution at 20 °C from 200 to 600 mPa. The chitin and chitosan materials were stored in a UNICO UN 650F mode box under an atmosphere of argon as they are hygroscopic. The chemicals 3-hexylthiophene (3HT), fluorene (F) and anhydrous FeCl_3_ were obtained from TCI and used without any further purification. Solvents, such as chloroform and methanol, were of analytical grade, purchased from Wako Pure Chemical Industry Ltd and used as received. Chloroform, the solvent used for the graft reaction, was dried by standing over 4A molecular sieves for 8 h and purged with argon gas for 20 min prior to use.

### 2.2 Measurements

UV-Vis spectroscopy was recorded using the diffuse reflectance measurement facility on a Jasco V-650 UV-Vis spectrometer. The fluorescence spectra were recorded at room temperature on a Jasco spectrofluorometer FP-8300 at an excitation wavelength of 360 nm. Thermogravimetric analysis (TGA) was conducted on a Hitachi Thermal Analysis System STA 7200 RV in air from 20 to 700 °C at a flow rate of 25 mL min^−1^ and a heating rate of 10 °C min^−1^. The ^1^H NMR spectra (400 MHz) and IR spectra were recorded on a Bruker Acsend 400 spectrometer and a Jasco FT/IR-480 Plus, respectively. The samples were dissolved in CDCl_3_ for the ^1^H NMR measurements. All samples including chitin/chitosan and grafted chitin/chitosan were crushed with KBr and then, the mixtures were compressed to form a pellet for the IR measurements. X-ray diffraction (XRD) patterns were recorded using Cu-Kα radiation (X-ray wavelength: 1.5418 Å) in steps of 0.02° over the 2*θ* range of 5–70° on a Rigaku Smartlab diffractometer equipped with a D-tex detector. Transmission electron microscopy (TEM) images were observed using a JEOL JEM-2100F microscope. Energy dispersive X-ray (EDX) mappings and line scan spectra were recorded on an Oxford INCA Energy TEM 250. For the TEM EDX measurements, 10 μL of a methanol suspension of the crushed samples were subjected to ultrasonication for 10 min and then deposited onto the TEM copper grids. Finally, the TEM copper grids were allowed to dry under ambient conditions. Gel permeation chromatography (GPC) was measured on a system equipped with a Jasco PU-2080 Plus pump and a Jasco RI-2031 plus intelligent RI detector. Chloroform served as the polymer solvent and the eluent in an equilibrated system at 40 °C. The quantum yields were analyzed on a Hamamatsu UV-NIR absolute PL quantum yield spectrometer. The samples were also compressed to form a film with thickness of 3 mm for the quantum yield measurement. Five replicates were performed for each sample. For a set of five measurements (*x*_1_…*x*_5_), the mean (*x̄*) was given by 
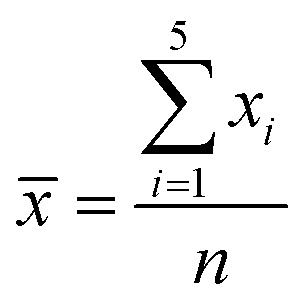
 and 
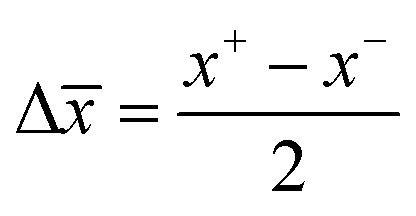
; where *x*^+^ and *x*^−^ are the highest and lowest values in the set, respectively and the results are reported as *x̄* ± Δ*x̄*.

### 2.3 Grafting and sample preparation

All reactions in this study were carried out in an oven-dried Schlenk flask with a stopcock under an argon atmosphere. Initially, 0.2 g of chitin and 0.4 g of FeCl_3_ were dispersed in 7 mL of chloroform using a magnetic stirrer. After the mixture was subjected to ultrasonication for 20 min, the suspension of chitin and FeCl_3_ in chloroform was cooled to 0 °C in an ice bath. A chloroform solution (3 mL) of 3HT (100 mg, 0.6 mmol) was poured to the magnetically stirred suspension of chitin and FeCl_3_. The reaction was conducted for 2 h at 0 °C under an argon atmosphere. The stoichiometric ratio of 3HT to FeCl_3_ was 1 : 4. The reaction was terminated by adding methanol. The obtained product was washed with methanol using a Soxhlet extraction apparatus to remove any residual FeCl_3_ and then, it was extracted with chloroform to eliminate the free poly(3-hexylthiophene) (P3HT) homopolymer. Grafting fluorene and the copolymer of fluorene and 3-hexylthiophene to chitin and chitosan was conducted using a similar procedure to that described above. Finally, the final products were dried under vaccum for 12 h. The grafted chitin/chitosan are hereafter referred as (3HT/F-*a*/*b*)-*g*-chitin and (3HT/F-*a*/*b*)-*g*-chitosan, where *a*/*b* represents the monomer ratio of 3HT and F used in the polymerization reaction.

## 3. Results and discussion

### 3.1 Photographic images of grafted chitin and chitosan

**Fig. 1:**
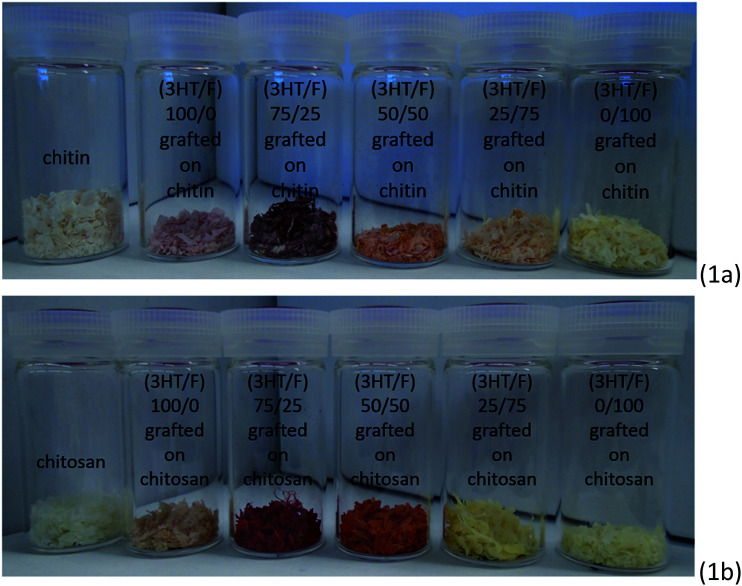
Photographic images of the copolymer of (3HT and F) grafted to (a) chitin and (b) chitosan.

In this study, a series of copolymers composed of 3HT and F with varying monomer feeds were employed as the chemical modifiers for the surface modification of polysaccharides (chitin and chitosan). It is worth noting that the resultant polysaccharides treated with these random copolymers exhibited visible multicolor (as shown in Fig. 1). Clearly, the different color appearance of the grafted chitin and chitosan samples changed noticeably by adjusting the fluorene content in the copolymer.

Chitin and chitosan are remarkably insoluble in water and most organic solvents. However, chitosan can dissolve well in a dilute aqueous acidic solution (pH ≤ 6) at various temperatures, while chitin is readily soluble in aqueous alkali solvents through a freeze-thawing process.^1,2,40,41^ Fig. S1 (ESI†) shows 20 mg of neat chitosan and (3HT/F-50/50)-*g*-chitosan in 1 mL CH_3_COOH (0.17 M) after being stirred for 24 h at 23 °C. As shown in Fig. S1,† neat chitosan is highly soluble in 1 mL 0.17 M CH_3_COOH. However, (3HT/F-50/50)-*g*-chitosan was insoluble in this solution. The insolubility of (3HT/F-50/50)-*g*-chitosan can be explained by the presence of the (3HT/F) copolymer on the chitosan.

### 3.2 FT-IR

FT-IR spectrometer, a simple and effective analytical instrument was utilized to identify the chemical bonding information and detect the functional groups of chitin/chitosan before and after their modification with the conjugated copolymers. The FT-IR spectra of the extracted P3HT, PF and (3HT/F) copolymers are shown in Fig. S2,† while the FT-IR spectra of the polysaccharides and modified polysaccharides are shown in Fig. S3.† After surface modification of the polysaccharides with the conjugated copolymers, the FT-IR spectra of the grafted polysaccharides (including chitin and chitosan) exhibited a new strong peak at around 770 cm^−1^, which proves that the grafting reaction took place on the surface of the modified polysaccharides (detailed analyses are provided in the ESI†).

### 3.3 GPC

The self-homopolymerization of 3HT and F occurred concurrently with the graft polymerization to chitin/chitosan during the grafting reactions of the conjugated copolymers to chitin/chitosan. The ungrafted 3-hexylthiophene and fluorene polymers were extracted from the grafted products using chloroform. After modification with the conjugated polymers, both the modified chitin and chitosan were not soluble in any solvent although all suggested methods were applied.^1,2,40,41^ Therefore, in this study, the molecular weights of the extracted copolymers were considered as the molecular weights of the graft copolymers on the surface of chitin/chitosan. The characterization of the extracted copolymers from the grafted chitin are similar to those extracted from grafted chitosan. As shown in Table S1,† (3HT/F-0/100)-*g*-chitin (entry 1) and (3HT/F-0/100)-*g*-chitosan (entry 6) have the lowest molecular weight, while (3HT/F-100/0)-*g*-chitin (entry 5) and (3HT/F-100/0)-*g*-chitosan (entry 10) have the highest molecular weight. In addition, with a decrease in the fluorene (F) monomer content, the molecular weight of the grafted (3HT/F) copolymer to chitin and chitosan increased (from entry 2 to entry 4 for chitin and from entry 7 to entry 9 for chitosan, respectively). These results can be explained based on the difference in the oxidation potential between 3HT and F.^42^ The oxidation potential of 3HT was lower than that of F^42^ and therefore, the oxidative polymerization of 3HT was easier than that of F. Thus, the molecular weight of (3HT/F-100/0)-*g*-chitin/chitosan is higher than that of (3HT/F-0/100)-*g*-chitin/chitosan. On varying the monomer feeds of 3HT/F from 100/0 to 0/100, the oxidation potentials of the copolymers possibly increased with the increase in F concentration, which resulted in a reduction of the molecular weight of the copolymers grafted on the modified polysaccharides.

### 3.4 Optical properties – UV-vis spectra, fluorescence spectra and quantum yields

**Fig. 2:**
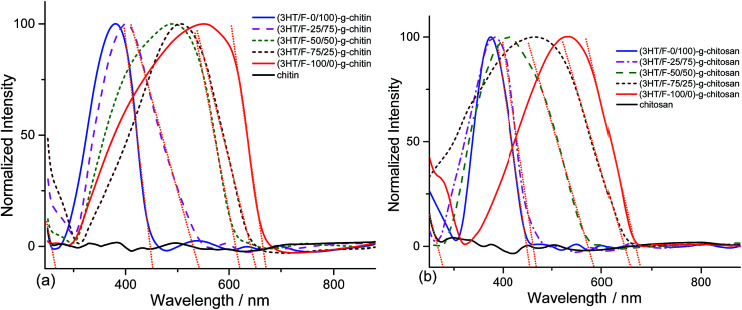
UV-Vis spectra of (a) chitin and grafted chitin; (b) chitosan and grafted chitosan.

Fig. 2 shows the UV-Vis spectra of chitin and chitosan grafted with the (3HT/F) copolymers. As shown in Fig. 2a and b, there are no absorption peaks observed in the range of 240–880 nm for neat chitin and chitosan, respectively. In contrast, strong absorption bands at around 250–650 nm appeared for the (3HT/F)-*g*-chitin and (3HT/F)-*g*-chitosan samples. These peaks can be assigned to the π–π* transitions from P3HT, PF and the copolymer of P3HT and PF. The maximum absorption peaks of (3HT/F-0/100)-*g*-chitin and (3HT/F-0/100)-*g*-chitosan are located at 379 and 372 nm, respectively, which are attributed to PF, while the maximum absorption peaks of (3HT/F-100/0)-*g*-chitin and (3HT/F-100/0)-*g*-chitosan are observed at 553 and 565 nm, respectively, which belong to P3HT.^32^ When the F content was increased from 25 to 75 in both the grafted chitin and chitosan samples, the maximum absorption peaks shifted to shorter wavelengths (as shown in Fig. 2 and Table 1). The shift in the *λ*^absorption^_max_ observed for the modified polysaccharides was the result of the random copolymer composed of 3HT and F with different monomer compositions, which was built-up on the surfaces of the polysaccharides. With the presence of the conjugated copolymers on the polysaccharides surface, the entire visible spectra range can be observed in the modified polysaccharides. The optical band gap of the grafted chitin/chitosan products were determined from the onset of absorption by finding the intersection point between the tangent line to a curve and the *x*-axis (as shown in Fig. 2). The maximum absorption wavelength, the onset absorption wavelength and optical band gap are summarized in Table 1. The band gap energy of chitin and chitosan are 4.66 and 4.45 eV, respectively. The presence of the conjugated copolymer of 3HT and F causes a decrease in the band gap energy of chitin and chitosan and the resultant *E*^op^_g_ (optical band gap) varied between 1.8 and 2.7 eV. The change in the band gap could originate from the F unit, which is in conjunction with the 3HT unit in the copolymers, which resulted in the extended conjugation length of the copolymer backbone on chitin/chitosan.

**Table 1:** The optical properties and band gap energy of the grafted chitin and chitosan samples

Samples	*λ* ^absorption^_max_ (nm)	*λ*_onset_ (nm)	*E* ^op^_g_ (eV)	*λ* ^emission^_max_ (nm)
Chitin	—	266	4.66	—
(3HT/F-0/100)-*g*-chitin	379	452	2.74	560
(3HT/F-25/75)-*g*-chitin	402	541	2.29	572
(3HT/F-50/50)-*g*-chitin	490	621	1.99	608
(3HT/F-75/25)-*g*-chitin	509	652	1.90	659
(3HT/F-100/0)-*g*-chitin	553	670	1.85	672
Chitosan	—	278	4.45	—
(3HT/F-0/100)-*g*-chitosan	372	454	2.73	556
(3HT/F-25/75)-*g*-chitosan	383	469	2.64	574
(3HT/F-50/50)-*g*-chitosan	413	584	2.12	605
(3HT/F-75/25)-*g*-chitosan	468	657	1.88	627
(3HT/F-100/0)-*g*-chitosan	565	676	1.83	653

**Fig. 3:**
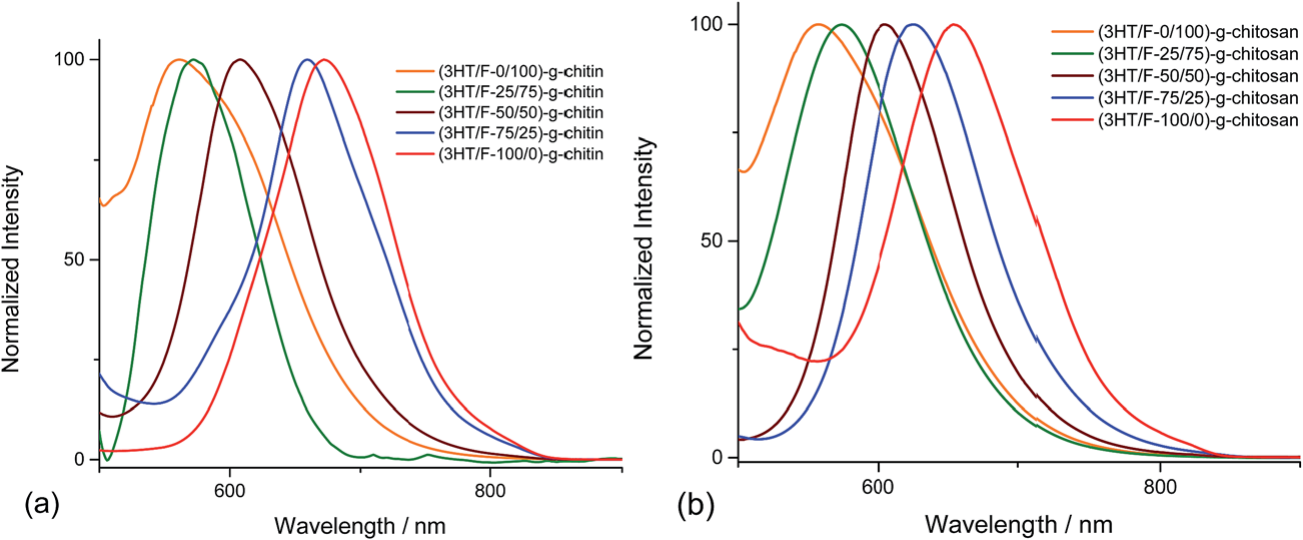
Fluorescence spectra of the (3HT/F) copolymers grafted to (a) chitin and (b) chitosan.

The fluorescence spectra of the (3HT/F)-*g*-chitin/chitosan samples display different emission bands in the visible-light region depending on the 3HT/F feed ratio. The emission spectra of the (3HT/F)-*g*-chitin/chitosan samples were recorded after excitation at 360 nm. As shown in Fig. 3, all the samples show a single emission in the wavelength range from 500 to 700 nm. The maximum emission peaks of all the grafted products are summarized in Table 1. The *λ*^emission^_max_ of (3HT/F-100/0)-*g*-chitin and (3HT/F-100/0)-*g*-chitosan are 672 and 653 nm, respectively. The presence of the F units in the (3HT/F) copolymer grafted to chitin and chitosan led to a blue-shift in the emission peak from about 650 nm to 550 nm (as shown in Fig. 3 and Table 1). The blue-shift in the luminescence spectra obtained for (3HT/F)-*g*-chitin/chitosan can be ascribed to the rigid biphenyl unit of PF, which results in a large band gap with efficient blue emission.^43^ Although a variety of copolymerizations composed of two conjugated monomers have been recently reported,^43^ this is the first time random copolymers derived from polyfluorene were grafted to polysaccharides, which caused the modified polysaccharides to emit colors spanning the entire visible-light range including red, green and blue. In summary, the fluorescence of the grafted chitin/chitosan was a response to the change in the feeding ratio between the 3HT and F units. In other words, the fluorescence of the grafted chitin/chitosan can be chemically manipulated by introducing of a series of 3HT/F copolymers on the surface of chitin/chitosan.

**Fig. 4:**
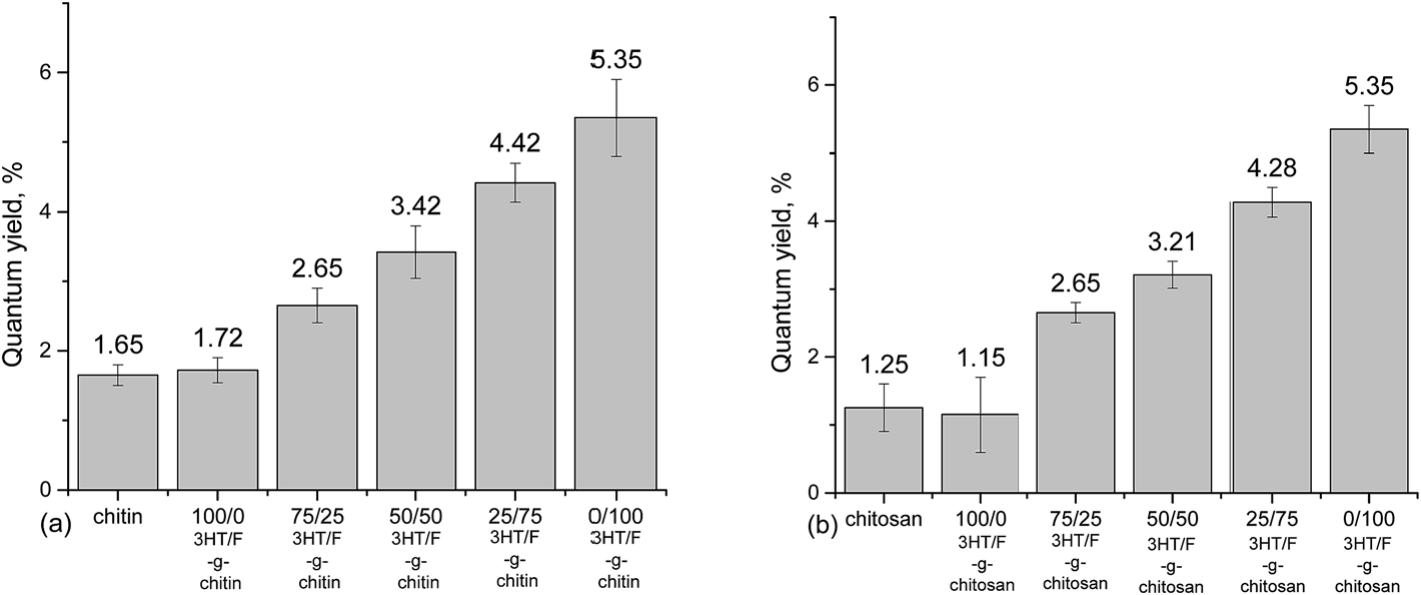
Quantum yields of (a) chitin and grafted chitin; (b) chitosan and grafted chitosan.

An absolute determination method^44,45^ was used to record the fluorescence quantum yield of chitin/chitosan and grafted chitin/chitosan containing the (3HT/F) copolymers. The quantum yield values of neat chitin and chitosan are not zero, which is still an obscurity to us until now. However, using the same measuring instrument, used under the same conditions, although neat chitin/chitosan and the modified chitin/chitosan showed a slight difference in quantum yield value, which increased by only 4%, the effects of the conjugated polymers on the quantum yield of modified chitin and chitosan were recognized. As shown in Fig. 4, the increase in the fluorene content in the (3HT/F) copolymer grafted to chitin/chitosan resulted in an increase in the quantum yield values of the grafted chitin/chitosan products. The quantum yield of chitin/chitosan and grafted (3HT/F-100/0) to chitin/chitosan are the lowest, while (3HT/F-0/100) grafted to chitin/chitosan has the highest quantum yields. These data agree very well with those obtained in our previous report.^32^ During the characterization of the P3HT and PF copolymers, the quantum yield determined using the relative method also increased with an increase in the fluorene content. In summary, the possible shift in both the absorption and emission bands observed for the grafted polysaccharides can be ascribed to the varying units of 3HT and F composition on the surface of the modified polysaccharides. Furthermore, with its highly fluorescent nature, the presence of the F units helps to tune the emission and results in an increased quantum yield. In short, the optical changes observed for the polysaccharides are generally related to the presence of the conducting polymers, which are attached on the modified polysaccharides surface.

### 3.5 Thermogravimetric analysis

**Fig. 5:**
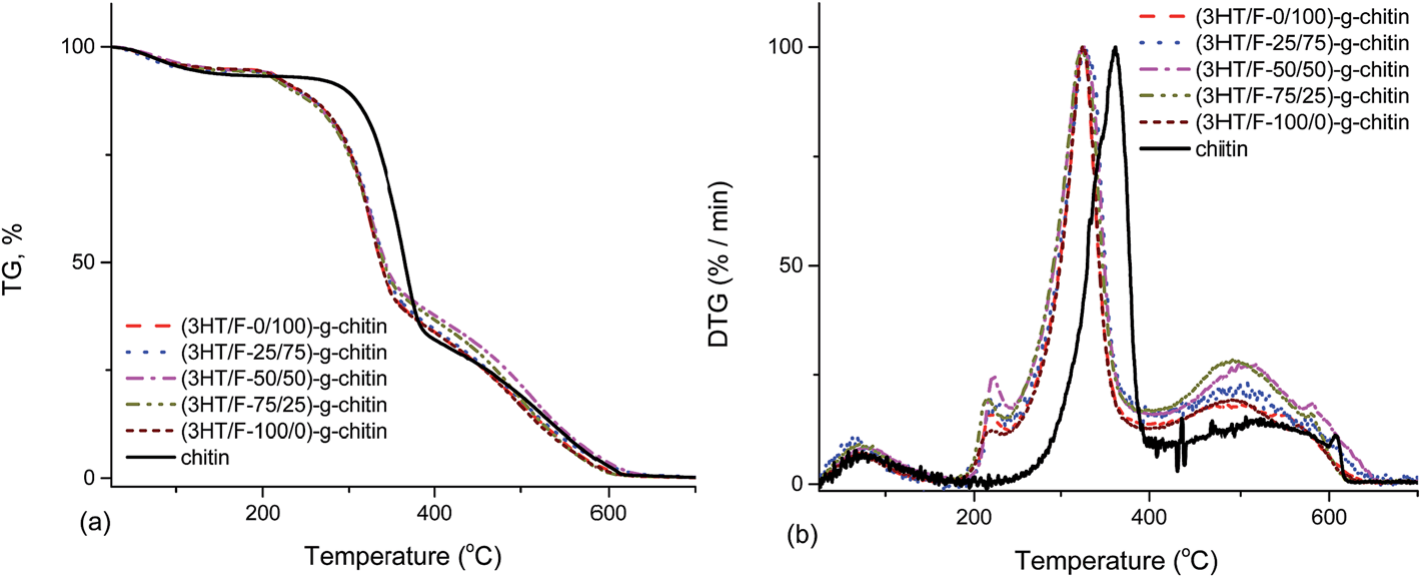
Thermogravimetric analysis (TGA) (a) and derivative thermogravimetric (DTG) analysis (b) curves of chitin and modified chitin.

Fig. 5 shows the TGA and DTG curves of chitin and the (3HT/F)-*g*-chitin samples. As shown in Fig. 5, the TGA curve of chitin shows two stages of decomposition in an air atmosphere. The first stage of degradation of chitin started from 245 to 389 °C with a weight loss from 7 to 67%. The second stage of decomposition of chitin occurred from 389 to 624 °C with a weight loss between 67 and 99%. The (3HT/F) copolymers grafted to chitin also show the two steps observed in the decomposition. However, all the grafted chitin samples began to decompose at a lower temperature than that of chitin. In detail, all the grafted chitins show an initial decomposition temperature of 206 °C. The difference in the thermal decomposition properties of these samples can be observed clearly from the DTG curves (Fig. 5b). In the DTG curves, there are two peaks belonging to the two-step degradation. The high temperature peak is considered as a measure of the thermal stability. In detail, the temperature peak of chitin was 363 °C, while it was 324 °C for the (3HT/F)-*g*-chitin samples. As a result, the thermal stabilities of (3HT/F)-*g*-chitin were lower by 39 °C than that of chitin.

**Fig. 6:**
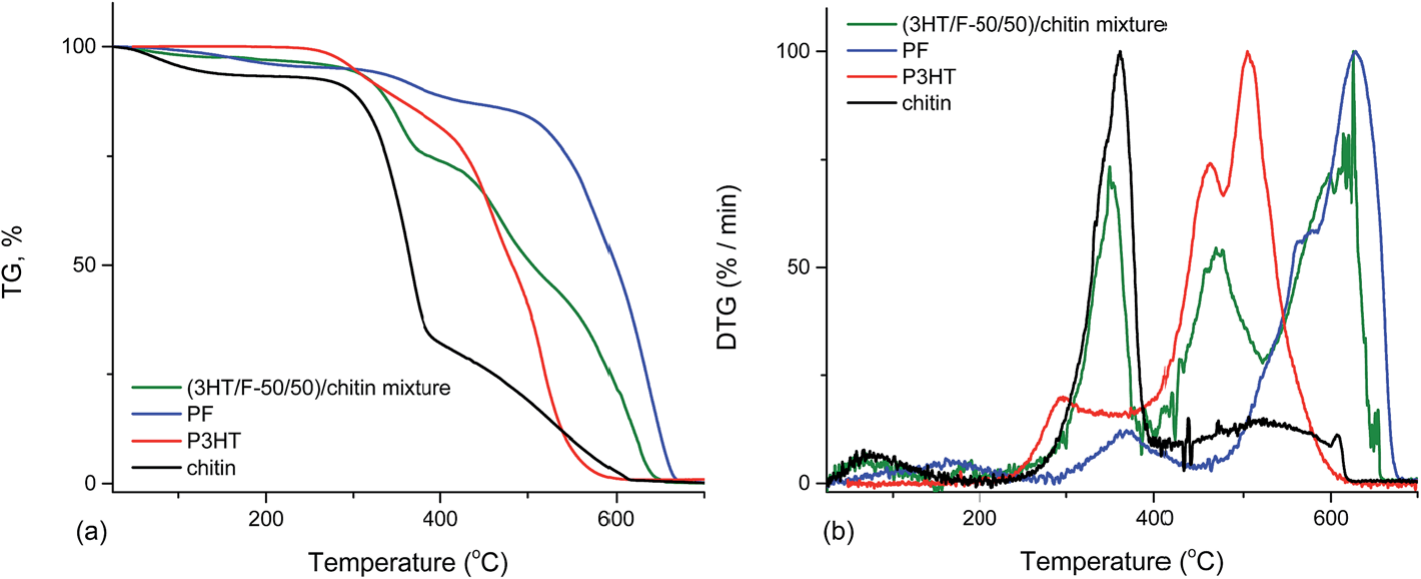
Thermogravimetric analysis (TGA) (a) and derivative thermogravimetric (DTG) analysis (b) curves of PF, P3HT, chitin, and (3HT/F)/chitin mixture.

The thermal stabilities of (3HT/F-50/50)-*g*-chitin and a simple mixture of (3HT/F-50/50) copolymer and chitin (namely (3HT/F)/chitin mixture) were compared. As shown in Fig. 6, the P3HT and PF homopolymers showed higher stabilities when compared to chitin itself. The DTG peak temperature is considered as a measure of the thermal stability. The DTG peak of chitin was 361 °C, while the DTG peaks of P3HT and PF were 505 and 629 °C, respectively. The TGA curve of the (3HT/F)/chitin mixture shows three stages of decomposition. The first stage of decomposition corresponds to the thermal degradation of chitin from 245 to 367 °C. The second and third stage of the degradation occurred from 371 to 444 °C and from 492 to 654 °C, respectively, which can be assigned to the decomposition of the (3HT/F) copolymer. The thermal stability of the (3HT/F-50/50)-*g*-chitin was different from the thermal decomposition properties of the (3HT/F)/chitin mixture. The thermal stability of (3HT/F)-*g*-chitin was slightly lower than that of chitin itself, while the thermal decomposition behavior of the (3HT/F)/chitin mixture was higher than that of chitin itself.

The decomposition behavior of chitosan and modified chitosan containing the (3HT/F) copolymers was similar to those of chitin and grafted chitin (as shown in Fig. S5†). The thermal stabilities of grafted chitosan and a simple mixture of (3HT/F-75/25) copolymer and chitosan (namely (3HT/F)/chitosan mixture) were also compared (Fig. S6†). The TGA results of the grafted polysaccharides and the mixture of polysaccharides and conjugated copolymers further supported the grafting of the conducting copolymers onto the polysaccharides.

The results from the TGA and DTG curves (Fig. 6 and S6†) can be explained based on the incompatibility of the constituent polymers, which causes the phase-separated structure in the polymer blend.^46^ The (3HT/F) copolymer was mixed with the polysaccharides, which is a combination of the polysaccharides and conjugated polymers, in which each component is individually distinct. Consequently, (3HT/F)/chitin and the (3HT/F)/chitosan mixture show separate temperature peaks, belonging to each constituent of the mixture. The observed difference between the thermograms of the grafted polysaccharides and (3HT/F)/polysaccharides mixtures indicate the interactions between the conjugated copolymers and polysaccharides.

In summary, the TGA and DTG curves indicated that (3HT/F) grafted to chitin/chitosan had lower thermal stability when compared to neat chitin/chitosan. In previous reports, the grafting vinyl monomers (methyl acrylate, methyl methacrylate, 2-hydroxyethylmethacrylate, *etc.*) to polysaccharides, such as cellulose, also resulted in a decrease in the thermal stability of the polysaccharide.^47–51^ The thermal stability of the polymer depends significantly on its crystallinity.^52^ Therefore, grafting the (3HT/F) copolymers to chitin/chitosan can increase the amorphous regions in chitin/chitosan, resulting in changing the crystallinity, which in turn, leads to a decrease in the thermal stability in the modified chitin/chitosan materials.

### 3.6 XRD

**Fig. 7:**
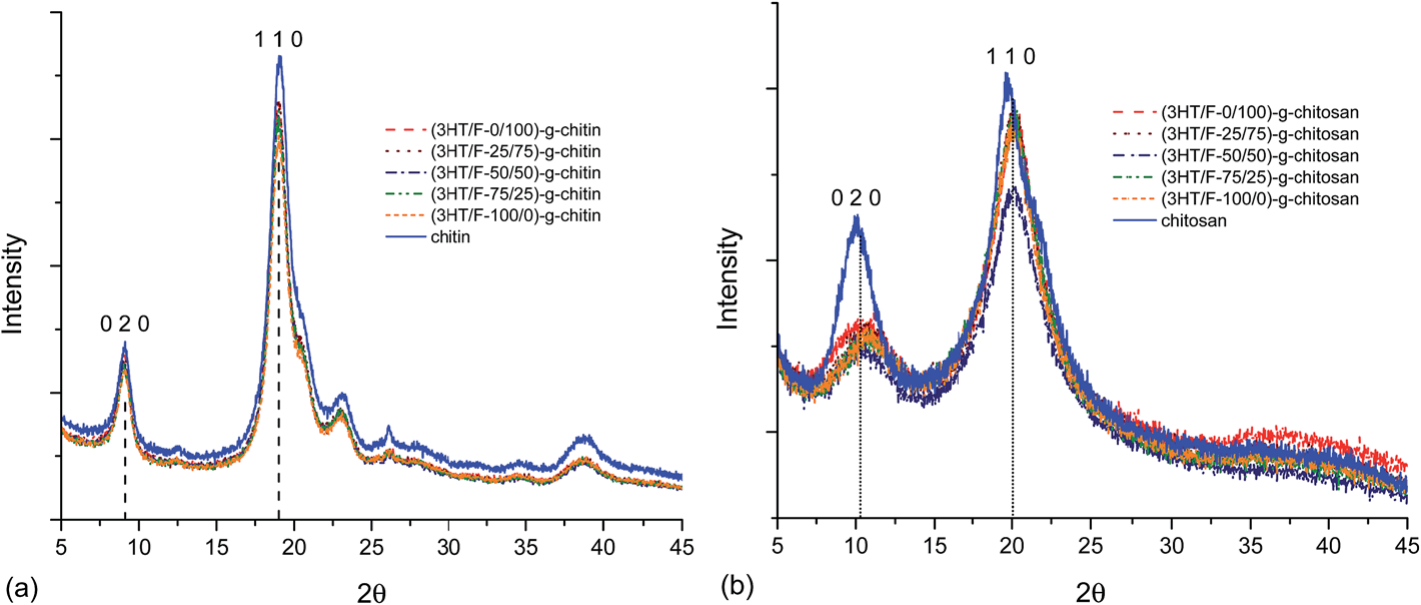
XRD patterns of (a) chitin and (3HT/F)-*g*-chitin and (b) chitosan and (3HT/F)-*g*-chitosan.

Various studies have reported the calculation of the degree of crystallinity of chitin and chitosan using X-ray measurements.^53–57^ There are two equations used for the determination the crystallinity index:

CrI_020_ = (*I*_020_ − *I*_am_)/*I*_020_ × 100 and CrI_110_ = (*I*_110_ − *I*_am_)/*I*_110_ × 100, where *I*_020_ is the maximum intensity of the crystalline peak from the (020) lattice diffraction, *I*_110_ is the maximum intensity of the crystalline peak from the (110) lattice diffraction and *I*_am_ is the intensity of amorphous diffraction at 2*θ* = 12.6°.^53–57^Fig. 7 shows the diffraction patterns of chitin/chitosan and (3HT/F) copolymer grafted to chitin/chitosan. The results obtained for the crystallinity index are summarized in Table S2.†

Neat chitin (Fig. 7a) shows three characteristic diffraction peaks at 9.1°, 19.1°, and 23.1°, corresponding to the 020, 110, 120 planes, respectively.^53–57^ After grafting the (3HT/F) copolymer to chitin, all the diffraction patterns coincide with those of neat chitin. However, the crystallinity index of all the grafted chitin samples decreased slightly when compared to the original chitin sample as summarized in Table S2.† The CrI_020_ and CrI_110_ are 63.1 and 85.6, respectively. After grafting the (3HT/F) copolymer to chitin, these values varied from 59 to 61% (CrI_020_) and from 83 to 84% (CrI_110_). The two characteristic diffraction peaks of neat chitosan were observed at 10.3° and 20.0°, which belong to the 020 and 110 planes, respectively (Fig. 7b).^53–57^ The diffraction patterns of the (3HT/F)-*g*-chitosan samples also show the two diffraction peaks of the 020 and 110 planes. As shown in Table S2,† the CrI_020_ values of the (3HT/F)-*g*-chitosan samples decrease significantly when compared to neat chitosan, while the CrI_110_ values of the (3HT/F)-*g*-chitosan samples slightly decrease. In summary, after grafting, the crystallinity index of chitin and chitosan are lower than that of neat chitin and chitosan. Grafting the (3HT/F) copolymers to chitin/chitosan introduce disorder in their crystalline structures and results in an increase in the amorphous regions. Furthermore, the thermal stability of the polymer depends mainly on the crystallinity.^52^ The XRD results are in agreement with the TGA results. Therefore, grafting the (3HT/F) copolymer to chitin/chitosan leads to lower thermal stabilities than chitin and chitosan.

### 3.7 TEM EDX

**Fig. 8:**
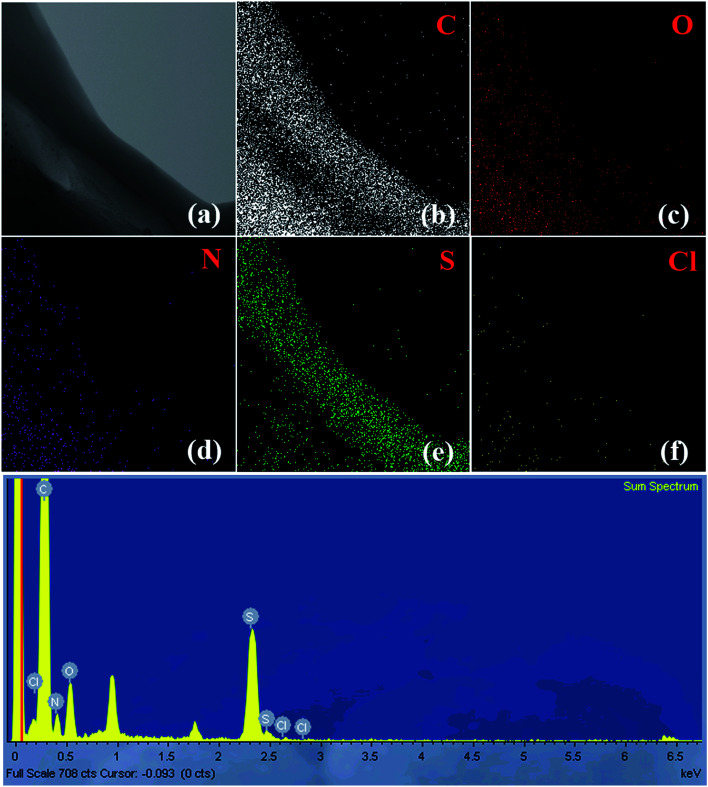
The EDX mapping images (a–f) and elemental spectrum of (3HT/F-50/50)-*g*-chitin.

Fig. 8 and S7† display the TEM-EDX analysis of the (3HT/F-50/50)-*g*-chitin and (3HT/F-50/50)-*g*-chitosan samples, respectively. The figures show the TEM images and EDX mappings, representing the distribution of the elements in the material. As shown in Fig. 8 and S7,† carbon, oxygen, nitrogen, chlorine, and sulfur are observed in both of the (3HT/F-50/50)-*g*-chitin and (3HT/F-50/50)-*g*-chitosan samples. The S observed in the EDX mapping was attributed to the heterocyclic compound of 3HT grafted to chitin and chitosan. No Fe element was detected in the final products, which indicates that all the FeCl_3_ was removed during the Soxhlet extraction process using methanol. The mass and atomic percentages of all elements in the grafted chitin and chitosan samples are summarized in Table S3.† However, the EDX technique only provides information on the chemical composition of elements with atomic numbers greater than three (*Z* > 3), so hydrogen cannot be detected using EDX.^58^ Thus, these values do not correctly account for the quantity of sulfur on the surface of chitin and chitosan.

## 4. Conclusions

In this article, a novel, facile, and simple oxidative procedure is reported for the fabrication of a conjugated copolymer layer comprised of P3HT and PF on the surface of chitin and chitosan. This approach is an interesting method to create chitin/chitosan materials with innovative optical properties. The desired blue-/red-shifted absorption and emission of grafted chitin/chitosan can be manipulated using different feed ratios of the 3HT/F units. The quantum yield of (3HT/F)-*g*-chitin/chitosan increased with an increase in the fluorene units. The crystallinity of chitin and chitosan decreased slightly after the graft polymerization of PF and P3HT. TEM EDX detected the sulfur atoms of the 3HT units on the grafted chitin/chitosan samples. The thermal stability of (3HT/F)-*g*-chitin/chitosan was slightly lower than that of chitin/chitosan, while the thermal decomposition behavior of a simple mixture of (3HT and F) and chitin/chitosan was higher than that of neat chitin/chitosan. The modified surface of chitin/chitosan using the (3HT/F) copolymer changed the degree of crystallinity in chitin/chitosan and thus decreased their thermal stabilities.

## Conflicts of interest

There are no conflicts to declare.

## Supplementary Material

RA-008-C8RA00287H-s001
