# Supplementary material for: Fluorescence control of chitin and chitosan fabricated via surface functionalization using direct oxidative polymerization
Source: RSC Adv. 2018 Feb 13;8(13):7005–13. doi: 10.1039/c8ra00287h (PMC9078334; doi:10.1039/c8ra00287h)
Supplement: RA-008-C8RA00287H-s001 [file RA-008-C8RA00287H-s001.pdf]

Electronic Supplementary Material (ESI) for RSC Advances.
This journal is © The Royal Society of Chemistry 2018

Supplementary Information

**Fluorescence control of chitin and chitosan fabricated by surface functionalization using directly oxidative polymerization**

Thien An Phung Hai, Ryuichi Sugimoto

*School of Environmental Science and Engineering, Kochi University of Technology,*

*Miyanokuchi, Tosayamada, Kami, Kochi 782-8502, Japan*

Correspondence to: Ryuichi Sugimoto, School of Environmental Science and Engineering, Kochi University of Technology, Miyanokuchi, Tosayamada, Kami, Kochi 782-8502, Japan

E-mail: an.phthien@gmail.com; sugimoto.ryuichi@kochi-tech.ac.jp

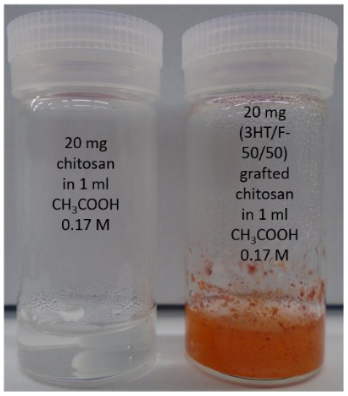


Figure S1. Photographic images of chitosan(left) and (3HT/F-50/50)-g-chitosan(right) in $CH_3COOH$ 0.17 M

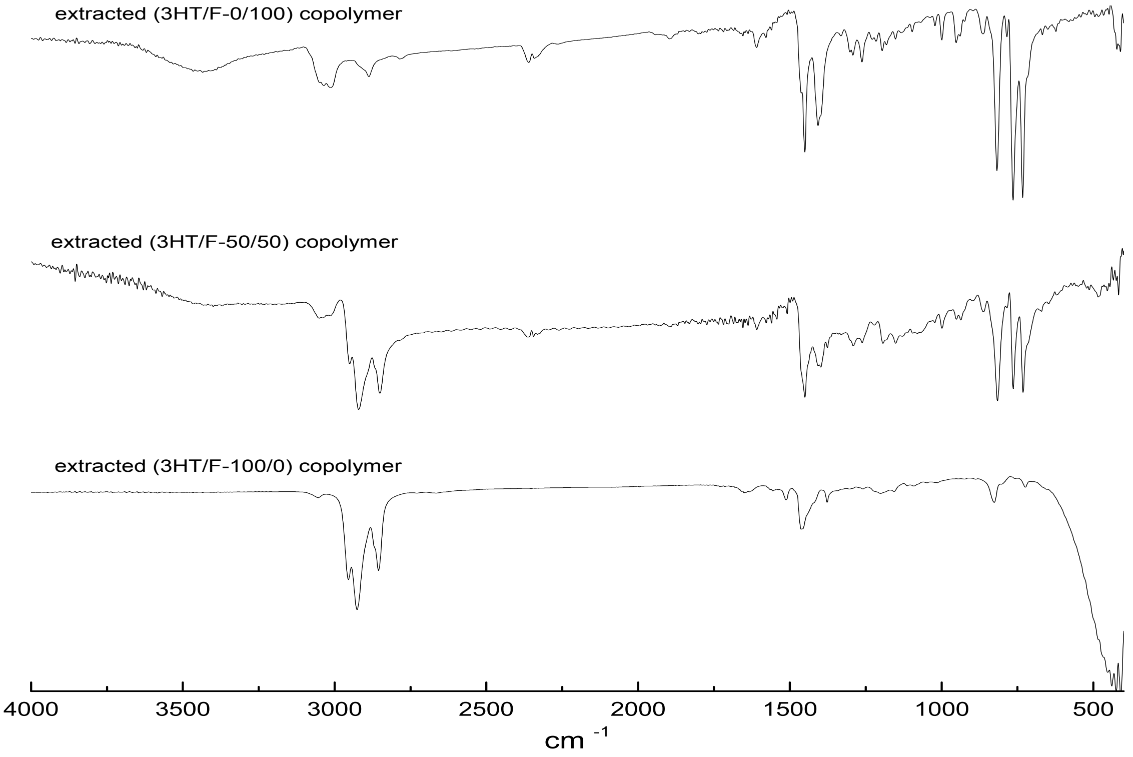


Figure S2. FT-IR spectra of extracted (3HT/F-100/0) copolymer (entry 5, Table S1), extracted (3HT/F-50/50) copolymer (entry 3, Table S1), and extracted (3HT/F-0/100) copolymer (entry 1, Table S1)

During the grafting reactions of copolymer composed of 3-hexylthiophene (3HT) and (fluorene) F to the surface of polysaccharides, the self-homopolymerizations of 3HT and F occurred concurrently with graft polymerization to chitin/chitosan. Figure S2 shows the FT-IR spectra of (3HT/F) copolymers extracted from grafted chitin by Soxhlet extraction apparatus using chloroform. The FT-IR spectra of (3HT/F) copolymers extracted from grafted chitosan show similar results to those extracted from grafted chitin. The weak peak observed in the range from 3000 to 3050 $cm^{-1}$ belongs to the aromatic C─H stretching vibrations while the cluster peaks at 3000─2800 $cm^{-1}$ are from the hexyl group of 3HT [1]. The C=C and C─C stretching mode from the aromatic of F and 3HT unit are seen at 1500 and 1450 $cm^{-1}$[1].

Previously, many investigations concerning the IR spectra of chitin characterization have been reported [2-5]. As shown in Figure S3a, virgin chitin shows the peak at 3561 $cm^{-1}$ represents the stretch of the OH group, and the bands at 3260 and 3097 $cm^{-1}$ are attributed to the vibration of NH group [4]. These peaks are seen at 2959, 2934, and 2880 $cm^{-1}$ belong to the asymmetric $CH_2$ stretching, $CH_3$ stretching, and CH symmetric [5]. These peaks at 1315 and 1375 $cm^{-1}$ belong to the $CH_2$ wagging, CH bending and $CH_3$ symmetric deformation. The amide I (inter-hydrogen bond between C=O with the N-H group and with the side chain group $CH_2OH$) and amide II (in-plane N-H bending and C-N stretching) bands are observed at 1660 and 1556 $cm^{-1}$, respectively [3-5]. The several peaks in the range of 1114 ─ 1216 $cm^{-1}$ correspond to C-O stretching and asymmetric bridge oxygen [6]. In grafted chitin samples, the bands at 3000─2800 $cm^{-1}$ from the characteristic of the hexyl group of 3HT unit could overlap with broad bands of various vibrations from the asymmetric $CH_2$ stretching, $CH_3$ stretching, and CH symmetric of chitin. Similarly, the C=C and C─C stretching mode from the aromatic of F and 3HT unit in the range of 1500 and 1450 $cm^{-1}$ are thought to overlap with amide II from chitin. However, a peak at 752 $cm^{-1}$ corresponds to CH out-of-plane vibration from the aromatic rings of 3HT and F unit [1, 7], became intense since grafting (3HT/F) copolymer to chitin.

As shown in the FT-IR spectra of pure chitosan from Figure S3b, the band from 3200-3600 $cm^{-1}$ belongs to the OH and NH stretching vibrations, the band observed from 2820 to 2950 corresponds to C─H stretching mode and the peak at 1660 $cm^{-1}$ is attributed to C=O stretching [2, 8-10]. The band at 3090-3030 $cm^{-1}$ from the υ ($C_\beta$─H) at the 4 position of 3HT unit [11] could be overlapped by the stretching vibrations of OH and NH groups. The $CH_3$ and $CH_2$ stretching vibrations from the hexyl group of 3HT unit at 3000-2800 $cm^{-1}$ may be also overlapped by the C─H stretching mode of chitosan. However, the CH out-of-plane vibration from the aromatic rings of 3HT and F unit [1, 7] appeared at 790 $cm^{-1}$, became sharper after grafting (3HT/F) to chitosan.

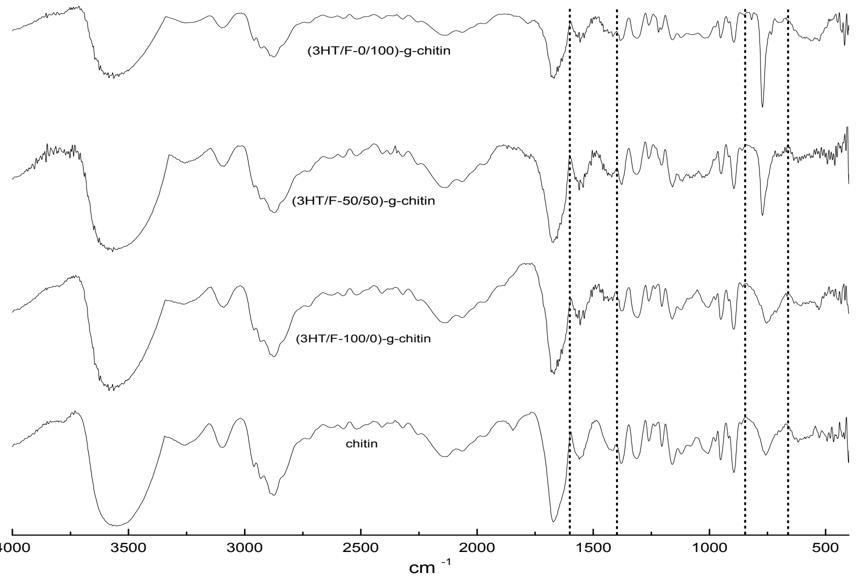


(S3a)

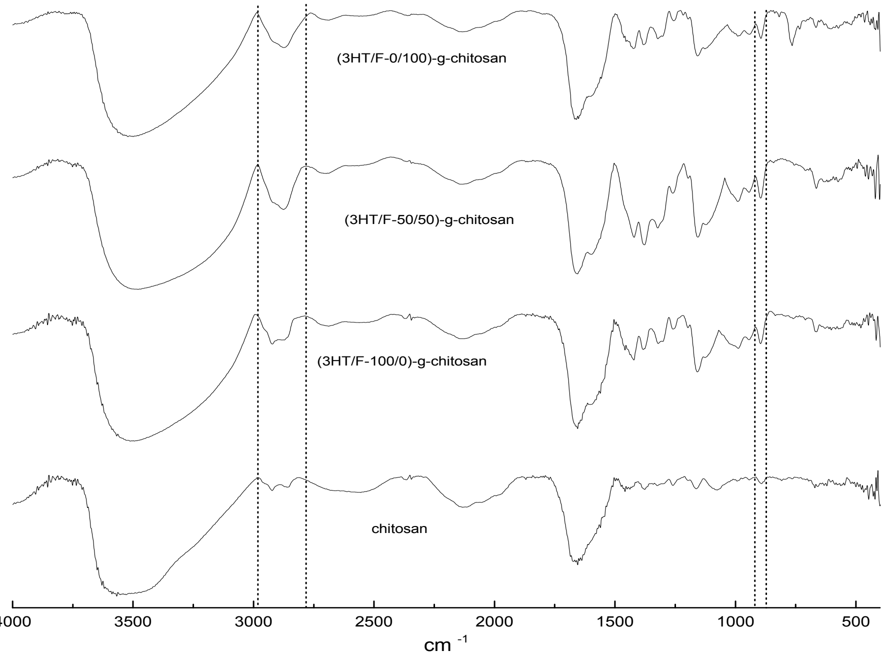


(S3b)

Figure S3. FT-IR spectra of (S3a) chitin and (3HT/F)-g-chitin; (S3b) chitosan and (3HT/F)-g-chitosan

Table S1: Characterization of grafted and extracted polymers

| Samples | Entry | 3HT/F | Gr % | Fluorene content in copolymer (**) | Molecular weight of graft copolymer (*) | |
|---|---|---|---|---|---|---|
| | | | | | $M_w$ ,kDa | PDI |
| (3HT/F) -g- chitin | 1 | 0/100 | 5.7 | 100 | 1.1 | 1.7 |
| | 2 | 25/75 | 4.7 | 69 | 3.0 | 1.9 |
| | 3 | 50/50 | 4.6 | 42 | 13.9 | 2.3 |
| | 4 | 75/25 | 5.1 | 18 | 25.1 | 3.0 |
| | 5 | 100/0 | 4.4 | 0 | 93.1 | 3.1 |
| (3HT/F) -g- chitosan | 6 | 0/100 | 5.4 | 100 | 0.8 | 1.4 |
| | 7 | 25/75 | 4.1 | 70 | 2.8 | 1.6 |
| | 8 | 50/50 | 5.5 | 40 | 11.5 | 2.9 |
| | 9 | 75/25 | 5.6 | 20 | 27.9 | 3.5 |
| | 10 | 100/0 | 5.8 | 0 | 86.1 | 3.3 |

(*) Determined by GPC; (**) Determined by $^1$H NMR; The graft ratio (Gr) was calculated by the following formulation: (A – B – C) / B x 100; where A is the weight of crude grafted polysaccharides product, B is the weight of original polysaccharides, and C is the weight of the extracted homopolymer or copolymer of fluorene and 3-hexylthiophene [12-14].

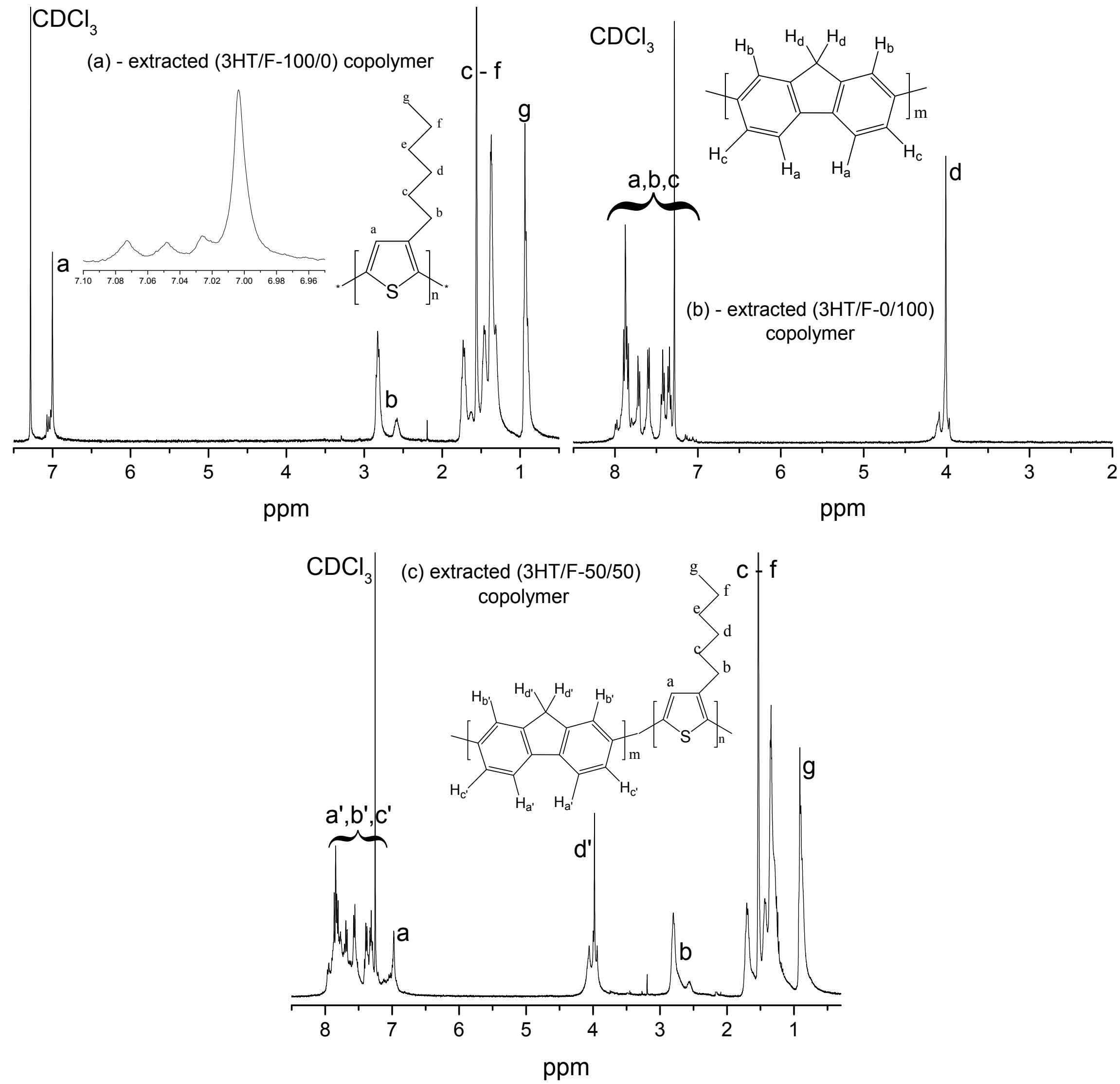


Figure S4. $^{1}H$ NMR spectra of (S4a) – extracted (3HT/F-100/0) copolymer (entry 5, Table S1), (S4b) – extracted (3HT/F-0/100) copolymer (entry 1, Table S1) and (S4c) – extracted (3HT/F-50/50) copolymer (entry 3, Table S1)

Figure S4 shows the $^{1}H$ NMR spectra of copolymers extracted from grafted chitin using chloroform. The methylene group of polyfluorene is identified by the signal at δ 4.0-4.2 ppm in Figure S4b while α-methylene proton in poly(3-hexylthiophene) is detected by the two peaks in the region δ 2.0-3.0 ppm in Figure S4a. The copolymer of 3HT and F in Figure S4c is identified by the two signal groups at δ 2.0-3.0 ppm (α-methylene of 3HT unit) and δ 4.0-4.2 ppm (methylene group of F unit). The F content in the copolymers can be calculated from the area scale of the peaks in two ranges [1]. The $^{1}H$ NMR spectra of copolymers extracted from grafted chitosan are similar to those extracted from grafted chitin.

Figure S5 shows the TG and DTG curves of chitosan and (3HT/F)-g-chitosan samples. As shown in the Figure S5a, the two stages of decomposition in an air atmosphere were shown in TG curves of chitosan. The first stage of decomposition of chitosan occurred from 253 to 339 $^{o}$C with weight loss from 11 to 46%. The second stage of degradation of chitosan started from 339 to 686 $^{o}$C where weight loss between 46 and 99%. After grafting with conjugated copolymers, all (3HT/F)-g-chitosan samples also showed the two steps in the decomposition. However, all grafted chitosan samples began the decomposition at lower temperature than that of chitosan. For instance, (3HT/F-25/75)-g-chitosan showed the first degradation from 224 to 325$^{o}$C and the second decomposition was in the range from 326 to 640$^{o}$C. The difference in the thermal decomposition properties of these samples can also be observed obviously from the DTG curves (Figure S5b). There are also two temperature peaks correspond to two step decompositions. The initial peak temperature is considered as a measure of the thermal stability. The peak temperature of chitosan is 318$^{o}$C while these values are 294$^{o}$C for (3HT/F)-g-chitosan samples. Therefore, the thermal stability of chitosan samples is higher by 24$^{o}$C than that of (3HT/F)-g-chitosan.

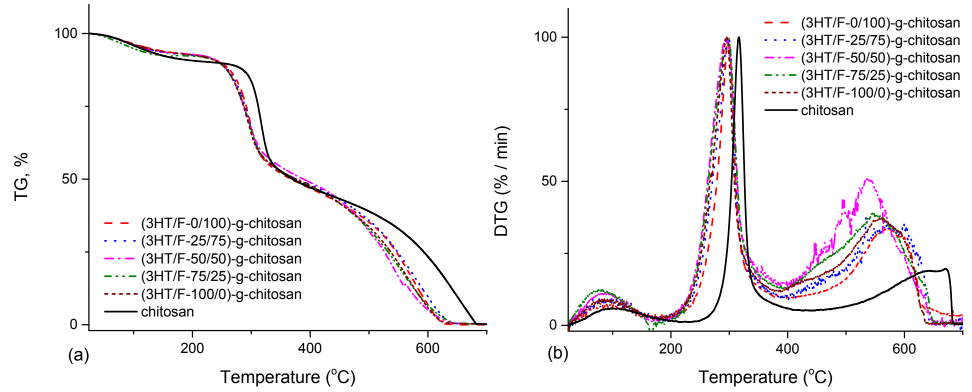


Figure S5. Thermogravimetric (TG) (a) and Derivative thermogravimetric (DTG) (b) curves of chitosan and grafting (3HT/F) copolymers to chitosan

The thermal stability of (3HT/F)/chitosan mixture shows the similar result to that of (3HT/F)/chitin mixture. As shown in Figure S6, the thermal stabilities of P3HT and PF are higher than that of chitosan itself. The DTG peak temperatures of P3HT and PF are 505 and 629 $^{o}$C, respectively. These values are considerably higher than that of chitosan (317 $^{o}$C). The thermal decomposition behavior of (3HT/F)/chitosan mixture also shows three DTG peak temperatures, the first peak at 317 $^{o}$C can be attributed to the degradation of the chitosan, the second and third peaks at 475 and 621 $^{o}$C belong to the decomposition of (3HT/F-75/25) copolymer chain. The thermal property of the (3HT/F)/chitosan mixture is also different from the decomposition behavior of the (3HT/F)-g-chitosan samples. These results are similar to the case of (3HT/F)-g-chitin and (3HT/F)/chitin mixture. It means the thermal stability of the

(3HT/F)-g-chitosan was slightly lower than that of chitosan itself, while the thermal decomposition behavior of the (3HT/F)/chitosan mixture was higher than that of chitosan itself.

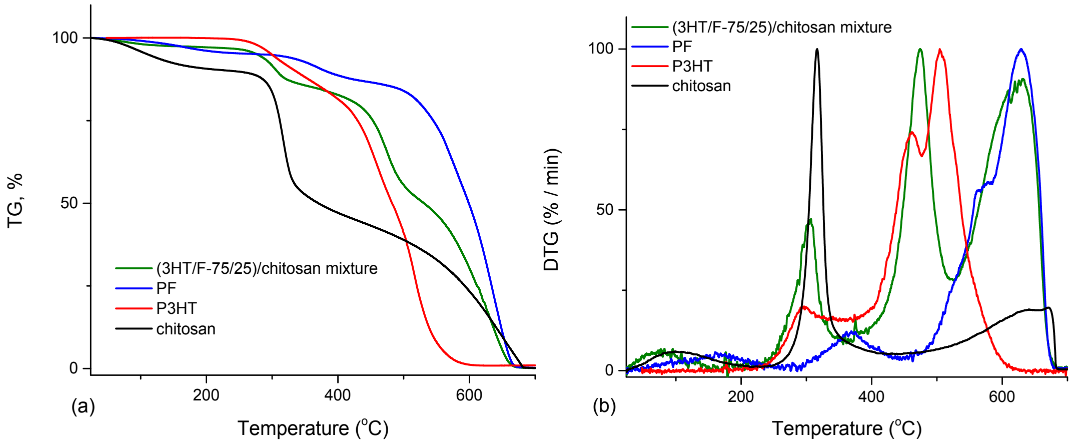


Figure S6. Thermogravimetric (TG) (a) and Derivative thermogravimetric (DTG) (b) curves of PF, P3HT, chitosan, and (3HT/F)/chitosan mixture

Table S2: Crystallinity index of chitin/chitosan and grafted (3HT/F) to chitin/chitosan

| Sample | Crystallinity Index ($CrI_{020}$) % | Crystallinity Index ($CrI_{110}$)% |
|---|---|---|
| chitin | 63.1 | 85.6 |
| (3HT/F-100/0)-g-chitin | 59.5 | 83.4 |
| (3HT/F-75/25)-g-chitin | 60.8 | 84.1 |
| (3HT/F-50/50)-g-chitin | 61.5 | 84.8 |
| (3HT/F-25/75)-g-chitin | 61.4 | 84.2 |
| (3HT/F-0/100)-g-chitin | 59.3 | 83.8 |
| chitosan | 40.1 | 56.1 |
| (3HT/F-100/0)-g-chitosan | 16.7 | 52.7 |
| (3HT/F-75/25)-g-chitosan | 15.8 | 52.1 |
| (3HT/F-50/50)-g-chitosan | 16.3 | 44.3 |
| (3HT/F-25/75)-g-chitosan | 17.5 | 51.8 |
| (3HT/F-0/100)-g-chitosan | 18.1 | 51.9 |

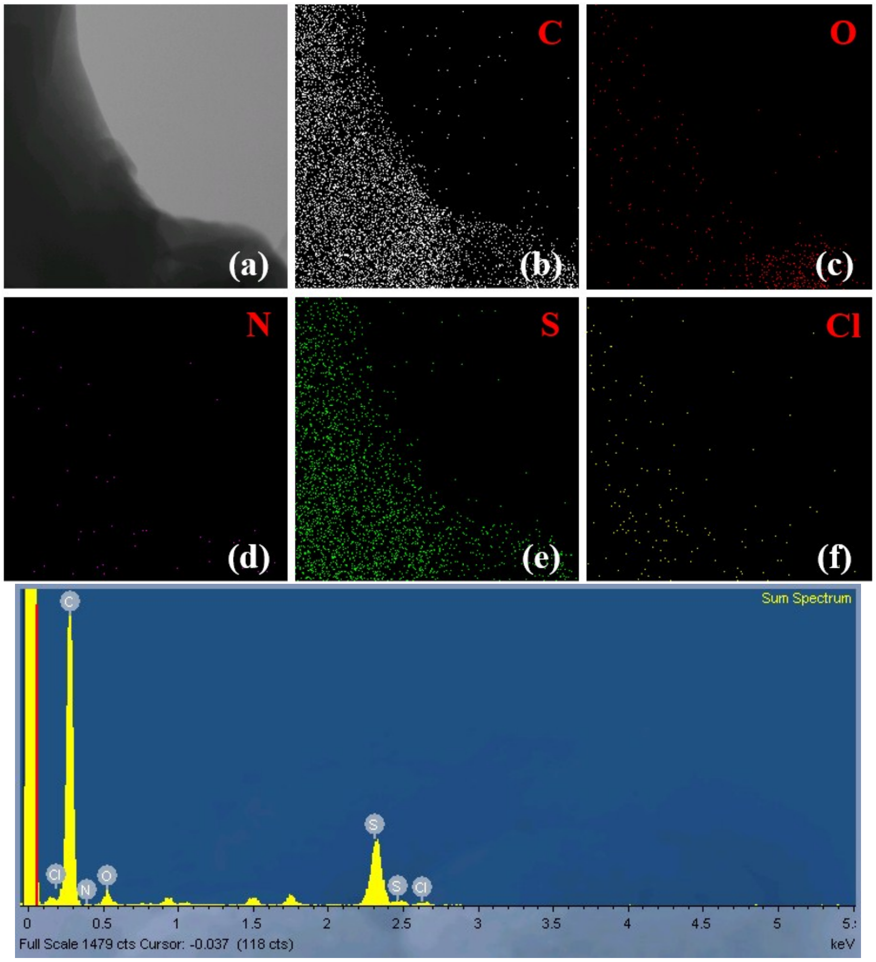


Figure S7. The EDX mapping images (a-f) and spectrum of (3HT/F-50/50)-g-chitosan

Table S3: The element composition of grafted chitin and chitosan

| Sample | Element | Weight% | Atomic% |
|---|---|---|---|
| (3HT/F-50/50)-g-chitin | C | 85.34 | 90.45 |
| | N | 3.74 | 3.40 |
| | O | 4.54 | 3.61 |
| | S | 6.29 | 2.50 |
| | Cl | 0.09 | 0.04 |
| (3HT/F-50/50)-g-chitosan | C | 84.69 | 92.42 |
| | N | 2.41 | 1.00 |
| | O | 4.29 | 3.69 |
| | S | 8.01 | 2.65 |
| | Cl | 0.60 | 0.24 |

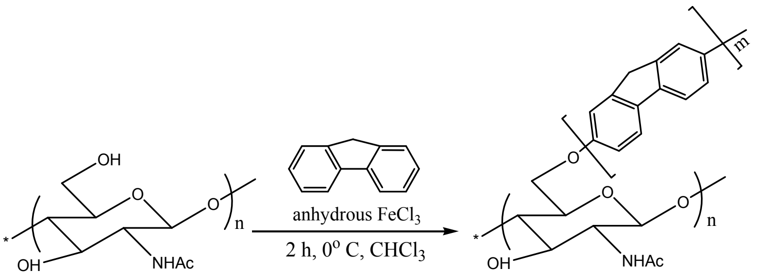


Scheme S1. Grafting of PF onto chitin

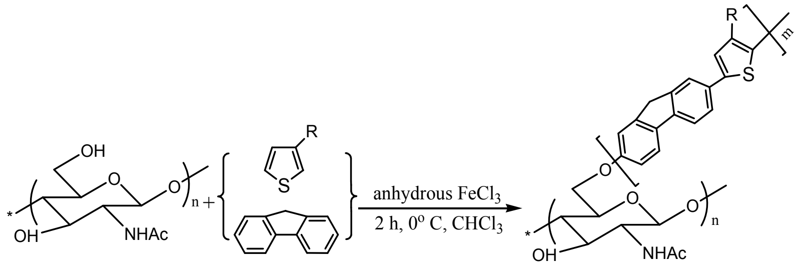


Scheme S2. Grafting of random copolymer of PF and P3HT onto chitin

Grafting P3HT, PF and (3HT/F) copolymers to polysaccharides are described in Schemes S1, S2 and S3. The proposed mechanism for grafting conjugated polymers on the surface of polysaccharide have been discussed in our previous reports [15-17]. In the first step, the radical formation on the chitin backbone may occur on the oxygen atom of the (-$CH_2OH$) group (Schemes S1, S2, and S3). At the same time, 3HT monomer were oxidized to be radical cation by $FeCl_3$ [18]. Initially, the oxidized 3HT monomer may couple with the free radical on the oxygen atom of the (-$CH_2OH$) group. In the propagation step, the $FeCl_3$ is continue to oxidize the 3HT molecule on the surface of chitin to form the radical cation. Then this radical cation on the surface of chitin is thought to couple together with another 3HT radical cation in solution. Repetition of this process could make P3HT layer to the surface of chitin. The termination may arise from the substitution of hydrogen of 3HT molecule with chlorine end group (Scheme 3) [19, 20]. It also explained the chlorine atom were detected in the (3HT/F)-g-chitin/chitosan by TEM EDX.

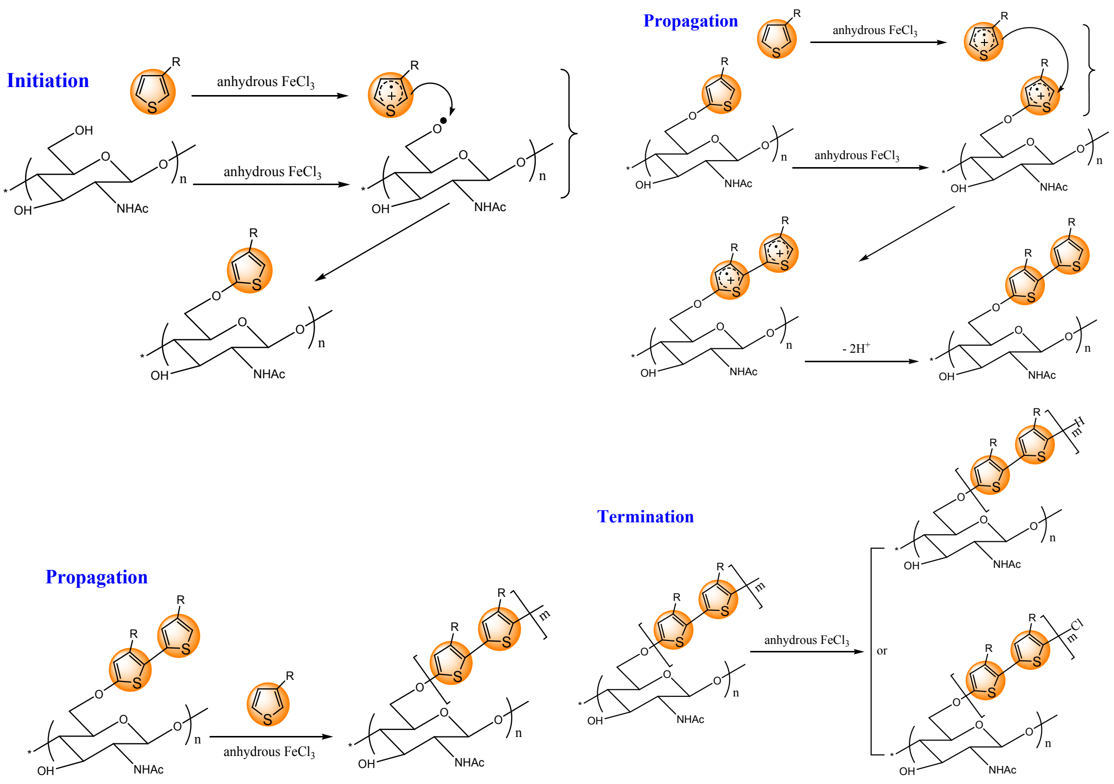


Scheme S3. Grafting of P3HT onto chitin

## References

[1] T. A. P. Hai, R. Sugimoto, *Polym. J.* **2016**, *48*, 1115.
[2] B. Focher, P. L. Beltrame, A. Naggi, G. Torri, *Carbohydr. Polym.* **1990**, *12*, 405.
[3] L. Ren, S. Tokura, *Carbohydr. Polym.* **1994**, *23*, 19.
[4] X. Chen, S. L. Chew, F. M. Kerton, N. Yan, *Green Chem.* **2014**, *16*, 2204.
[5] X. Chen, Y. Liu, F. M. Kerton, N. Yan, *RSC Adv* **2015**, *5*, 20073.
[6] M. Osada, C. Miura, Y. S. Nakagawa, M. Kaihara, M. Nikaido, K. Totani, *Carbohydr. Polym.* **2013**, *92*, 1573.
[7] G. Socrates, *John Wiley & Sons, Ltd* **2001**, *West Sussex, England*, 268.
[8] A. Umar, A. A. Naim, M. M. Sanagi, *Mater. Lett.* **2014**, *124*, 12.
[9] P. Negrea, A. Caunii, I. Sarac, M. Butnariu, *Dig. J. Nanomater. Biostruct.* **2015**, *10*, 1129
[10] A. G. Yavuz, A. Uygun, V. R. Bhethanabotla, *Carbohydr. Polym.* **2009**, *75*, 448.
[11] T. Hirai, Y. Nagae, K. L. White, K. Kamitani, M. Kido, T. Uchiyama, M. Nishibori, Y. Konishi, K. Yokomachi, R. Sugimoto, K. Saigo, T. Ohishi, Y. Higaki, K. Kojio, A. Takahara, *RSC Adv* **2016**, *6*, 111993.
[12] N. Nishioka, N. Yoshida, *Polym. J.* **1992**, *24*, 1009.
[13] N. Nishioka, M. Yamaoka, H. Haneda, K. Kawakami, M. Uno, *Macromolecules* **1993**, *26*, 4694.
[14] N. Nishioka, K. Kosai, *Polym. J.* **1981**, *13*, 1125.
[15] T. A. P. Hai, R. Sugimoto, *Biomacromolecules* **2017**, *18*, 4011.
[16] T. A. P. Hai, R. Sugimoto, *Carbohydr. Polym.* **2018**, *179*, 221.
[17] T. A. P. Hai, R. Sugimoto, *Appl. Surf. Sci.* **2018**, *434*, 188.
[18] V. M. Niemi, P. Knuuttila, J. E. 0sterholm, J. Korvola, *Polym. Rep.* **1992**, *33*, 1559.
[19] Y. Liu, N. Nishiwaki, K. Saigo, R. Sugimoto, *Bull. Chem. Soc. Jpn.* **2013**, *86*, 1076.
[20] T. D. McCarley, C. O. Noble, IV, C. J. DuBois, Jr., R. L. McCarley, *Macromolecules* **2001**, *34*, 7999.
